# To the Medical Profession of Georgia, Florida and South Carolina

**Published:** 1907-03

**Authors:** 


### TO THE MEDICAL PROFESSION OF GEORGIA, FLORIDA AND SOUTH CAROLINA.

---

The medical profession of San Francisco lost its medical library, the San Francisco County Medical Society Library, in the first last spring. Most of the physicians also lost whatever private libraries they had succeeded in collecting. A committee (named below) has been appointed by the American Medical Association and by the Association of the American Physicians to collect and send books to San Francisco, both for the library and for private individuals when duplicate copies are sent on.

Will you send by freight to Dr. E. Bates Block, 723 Empire Bldg., Atlanta, Ga., any medical books of value or bound volumes of Journals which you can spare? Fairly recent editions of standard text books, foreign text books or bound Journals (French, German, and Italian), hospital reports, monographs of all sorts, books on special subjects old classics, e. g., Trousseau, Charcot), and the Sydenham Society publications are especially desired.

Acknowledgement of all that is received will be made through the Medical Journals, and the books will be packed and shipped as promptly as possible. Signed:

CHARLES L. DANA, Chairman, New York City.

Frank Billings, Chicago. George W. Crile, Cleveland.
E. Bates Block, Atlanta. W. E. Fischel, St. Louis.
J. A. Capps, Chicago. F. Forchheimer, Cincinnati.
T. D. Coleman, Augusta, Ga. Charles L. Greene, St. Paul.

---

The thirst following a hemorrhage from gastric ulcer is best relieved by small quantities of cocain in solution.—American Journal of Surgery.

